# Effects of Sodium-Glucose Cotransporter 2 Inhibitors on Lower eGFR Decline in Nondiabetic CKD Patients without Proteinuria

**DOI:** 10.34067/KID.0000000886

**Published:** 2025-06-19

**Authors:** Masaru Matsui, Takaaki Kosugi, Kosuke Tansho, Shunsuke Kitamura, Masatoshi Nishimoto, Keisuke Okamoto, Masahiro Eriguchi, Ken-ichi Samejima, Kazuhiko Tsuruya

**Affiliations:** 1 Department of Nephrology, Nara Medical University, Nara, Japan; 2 Department of Nephrology, Nara Prefectural General Medical Center, Nara, Japan

**Keywords:** CKD, SGLT2, SGLT2 inhibitors

## Abstract

**Key Points:**

- Sodium-glucose cotransporter 2 inhibitors (SGLT2is) significantly slowed chronic decline in eGFR in nondiabetic CKD patients without proteinuria.
- Propensity score–matched analysis confirmed that SGLT2i users had a significantly slower postinitial dip eGFR slope than nonusers.
- Subgroup analyses consistently supported the renoprotective effect of SGLT2is in this population.

**Background:**

Sodium-glucose cotransporter 2 inhibitors (SGLT2is) have shown promise as renoprotective agents, building on the success of renin-angiotensin system blockers. Although SGLT2is have been shown to slow renal deterioration in diabetic and nondiabetic CKD with proteinuria, it is unclear whether similar effects occur in nondiabetic, nonproteinuric CKD.

**Methods:**

This study used propensity score analysis to evaluate the effects of SGLT2is on changes in annual eGFR in nondiabetic CKD patients with trivial proteinuria (urinary protein–creatinine ratio <0.5 g/gCr) who were seen at the Nara Prefecture General Medical Center from January 1, 2019, to December 31, 2022. The study analyzed 362 nondiabetic patients with CKD, including 211 SGLT2i users and 151 nonusers, with a median age of 65 (53–73) years, median eGFR of 45 (35–53) ml/min per 1.73 m^2^, and median urinary protein–creatinine ratio of 0.15 (0.09–0.29) g/gCr.

**Results:**

Adjusted linear mixed-effects models showed that while the eGFR decline over a 3-year period (total) was similar between the two groups, there was a significantly smaller decline between 3 months after baseline and 2-year follow-up (chronic) in SGLT2i users compared with nonusers, with a difference of 1.23 (95% confidence interval, 0.43 to 2.02; *P* = 0.002) ml/min per 1.73 m^2^ per year. After propensity score matching, SGLT2i users exhibited significantly slower chronic decline than nonusers, with a difference of 1.50 (95% confidence interval, 0.63 to 2.36; *P* < 0.001) ml/min per 1.73 m^2^ per year. Subgroup analyses confirmed these findings.

**Conclusions:**

This study suggests that SGLT2is may slow eGFR decline in non-diabetic CKD patients with trivial proteinuria, supporting the potential use of SGLT2is as renoprotective agents in this population.

## Visual Abstract

**Figure:**
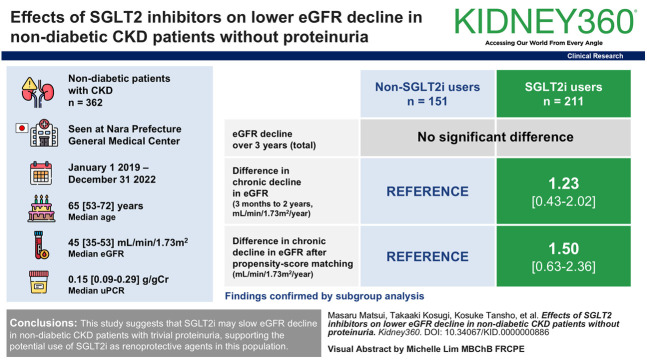

## Introduction

In the 2000s, renin-angiotensin system inhibitors (RASis) generated confirmed evidence as kidney protective agents in patients with proteinuric CKD.^1,2^ Despite large efforts, no breakthrough drugs emerged for CKD in the following 20 years, but this trend has evolved toward the prevention of CKD progression through early intervention of reducing BP and glucose and multidisciplinary education.^3–5^ Sodium-glucose cotransporter 2 inhibitors (SGLT2is) emerged as antihyperglycemic drugs by antagonizing sodium-glucose cotransporter 2 located in the early proximal renal tubule, resulting in a significant increase in urinary sodium and glucose excretion. The Empagliflozin Cardiovascular Outcome Event Trial in Type 2 Diabetes Mellitus Patients (EMPA-REG OUTCOME) trial demonstrated a SGLT2i significantly reduces cardiovascular and kidney events in patients with type 2 diabetes.^6,7^ Subsequently, cardiorenal protective effects on SGLT2is are verified in several clinical trials for diabetes.^8–10^

The Study to Evaluate the Effect of Dapagliflozin on Renal Outcomes and Cardiovascular Mortality in Patients With Chronic Kidney Disease (DAPA-CKD) trial first showed that similar beneficial kidney effects are seen in nondiabetic CKD patients with overt proteinuria.^11^ Dapagliflozin, however, did not significantly decrease the primary composite outcome among CKD patients with IgA nephropathy and ischemic and hypertensive kidney diseases with urinary albumin-creatinine ratio (UACR) <1000 mg/g.^12,13^ In the Study of Heart and Kidney Protection With Empagliflozin (EMPA-KIDNEY) trial, patients with CKD in empagliflozin versus placebo group have approximately 30% risk reduction of progression of kidney disease or death from cardiovascular causes in patients with more advanced CKD. Subgroup analyses have revealed that although patients with UACR <30 mg/g experienced a relatively low number of events, there were no significant difference between the empagliflozin and placebo groups among these patients.^14^

These results suggest it is not evident whether SGLT2is have beneficial effects on eGFR decline in nonproteinuric CKD. In this study, we therefore aimed to clarify beneficial effects of SGLT2is on annual eGFR slopes in nonproteinuric CKD patients without diabetes who received multidisciplinary education.

## Methods

### Patients

This hospital-based, retrospective, observational study initially included 519 nondiabetic patients with CKD who visited the Nara Prefecture General Medical Center, Japan, between January 1, 2019, and December 31, 2022, and for whom baseline data were available during this period. The following patients were excluded: 99 with urinary protein–creatinine ratio (UPCR) of ≥0.5 g/gcre, 12 with diabetes, three with AKI, 11 with missing data, and 21 lost to follow-up. In addition, 11 patients discontinued SGLT2is because of adverse events, such as fatigue and skin eruption. Finally, 211 SGLT2i users and 151 non-SGLT2i users were included in the analysis. Continuation or discontinuation of SGLT2is was determined with reference to entries in the electronic medical record at the time of eGFR evaluation. SGLT2is used in this study were dapagliflozin or empagliflozin at a dose of 10 mg/d.

At our institution, all patients with CKD routinely receive multidisciplinary education, which includes doctors, nurses, dietitians, and pharmacists who participate in medical treatment, patient education, diet consultation, and behavioral adjustment. At the time of the education, their baseline data, including clinical characteristics and laboratory findings, were collected through medical records and subsequent changes in eGFR were longitudinally followed in patients who initiated SGLT2i and those who did not. Our Hospital Ethics Board approved the study (No. 316). The research content is included on our department's web page.

### Study Outcomes

Our analysis was conducted using an on-treatment approach to assess the effectiveness of SGLT2i treatment in the SGLT2i group, and thus, patients who discontinued SGLT2is by death or adverse events were excluded in the study. The primary outcome was the difference of longitudinal eGFR changes between SGLT2i users and nonusers when adjusted for covariates. In both groups, eGFR levels were measured at the following five time points: 1 year before baseline data; at the visit of the hospital (baseline); and 3 months, 1 year, and 2 years after baseline data. For both SGLT2i users and nonusers, the baseline eGFR data were obtained at the time of the multidisciplinary education session. The eGFR was calculated using the Japanese equation.^15^ Furthermore, two eGFR trajectories were similarly expressed with a propensity score (PS) matching method to minimize differences between the two groups. To compare eGFR slopes between SGLT2i users and nonusers, we used total slope, defined as the mean eGFR changes from baseline to 2-year follow-up, and postinitial dip slope, defined as the mean eGFR changes from 3 months after baseline to 2-year follow-up.

### Statistical Analyses

All variables are expressed as medians (interquartile range). Differences between the groups were determined using Mann–Whitney *U* test or chi-squared test. A mixed-effects model with an unstructured variance–covariance structure was used to examine the longitudinal eGFR trajectory according to the use of SGLT2is, accounting for patient-level random effects. Factors included in the model were age, sex, baseline eGFR, eGFR 1 year before baseline, UPCR, body mass index, etiology of CKD, baseline use of RASis, and visit month before and after PS matching. PSs were calculated using multivariable logistic regression to estimate the probability of receiving SGLT2is. Demographics (age and sex), comorbidities and lifestyle (hypertension, dyslipidemia, body mass index, and current and previous smoker), laboratory tests (eGFR at baseline, eGFR 1 year before baseline, UPCR, and hemoglobin), etiology of CKD, and use or no use of RASis were included as covariates. Covariates were included in the PS model. PSs were then used to match SGLT2i users to nonusers 1:1 using a greedy nearest-neighbor matching algorithm.

Two-sided *P* values < 0.05 were considered statistically significant. STATA MP, version 17 software (Stata Corp., College Station, TX), were used to perform all statistical analyses.

## Results

### Baseline Characteristics

**Figure 1:**
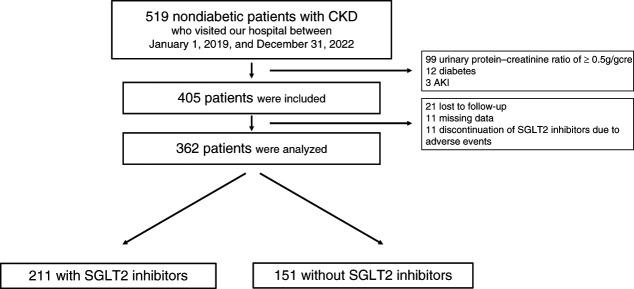
**Patient flow chart.** SGLT2, sodium-glucose cotransporter 2.

This study analyzed 211 SGLT2i users and 151 nonusers in nondiabetic CKD and UPCR <0.5 g/gcre (Figure 1). Baseline characteristics before and after PS matching are listed in Table 1. Before PS matching, SGLT2i users were significantly more likely to be male, have a higher body mass index level, and have a greater prevalence of GN and nephrosclerosis as underlying kidney diseases compared with nonusers. However, no significant differences were observed between the two groups in baseline characteristics after PS matching. Other laboratory findings are presented in Supplemental Table 1.

**Table 1:** Baseline characteristics before and after propensity score matching

Patient Characteristics	Before PS Matching		*P* Value	After PS Matching		*P* Value
	SGLT2i Users	Non-SGLT2i Users		SGLT2i Users	Non-SGLT2i Users	
No. of participants	211	151		110	109	
Age, yr	64 (53–72)	67 (52–76)	0.94	67 (57–72)	66 (52–75)	0.49
Sex (male), *No.* (%)	133 (63)	78 (52)	0.03	59 (54)	67 (61)	0.34
**Etiology of CKD, *No.* (%)**						0.14
Chronic GN	76 (36)	39 (26)	<0.001	26 (24)	33 (30)	
Nephrosclerosis	96 (45)	46 (30)		51 (46)	37 (34)	
Other	39 (18)	66 (44)		32 (29)	40 (37)	
Body mass index, kg/m^2^	24.2 (22.0–26.3)	23.1 (20.4–25.3)	0.002	24.1 (21.3–25.8)	23.2 (21.0–25.6)	0.19
Hypertension, *No.* (%)	132 (63)	85 (56)	0.23	64 (58)	66 (61)	0.78
Dyslipidemia, *No.* (%)	87 (41)	54 (36)	0.23	46 (42)	44 (40)	0.78
Smoker, *No.* (%)	45 (21)	48 (32)	0.14	26 (24)	33 (30)	0.36
eGFR 1 yr before baseline, ml/min per 1.73 m^2^	47 (36–57)	46 (37–53)	0.10	47 (40–53)	47 (35–56)	0.39
eGFR at baseline, ml/min per 1.73 m^2^	45 (33–55)	45 (36–53)	0.17	45 (38–53)	45 (33–55)	0.54
Proteinuria, g/gcre	0.15 (0.10–0.28)	0.16 (0.08–0.29)	0.51	0.13 (0.10–0.25)	0.16 (0.08–0.27)	0.74
Hemoglobin, g/dl	13.5 (12.5–14.6)	13.0 (11.7–14.1)	0.003	13.3 (12.5–14.4)	13.1 (12.0–14.3)	0.21
Renin-angiotensin receptor blocker, *No.* (%)	118 (56)	67 (44)	0.02	53 (48)	53 (49)	0.95
Urinary tract infection/genital infection, *No.* (%)	3 (1.4)	1 (0.6)	0.50	1 (0.9)	0 (0)	0.31

PS, propensity score: SGLT2i, sodium-glucose cotransporter 2 inhibitor.

### Difference in eGFR Slopes

**Figure 2:**
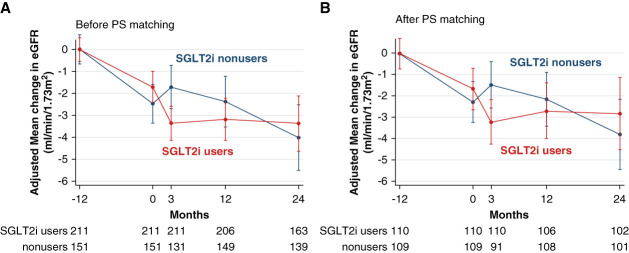
**Adjusted eGFR changes in SGLT2 users and nonusers before and after PS matching.** (A) Before PS matching (B) after PS matching. PS, propensity score; SGLT2i, sodium-glucose cotransporter 2 inhibitor.

Figure 2A showed the difference in adjusted mean eGFR changes between SGLT2i users and nonusers over 3-year periods. Before PS matching, mean changes in eGFR from baseline at 3 months were significantly different between SGLT2i users and nonusers (*P* < 0.001); it corresponded to a 2.9% (95% confidence interval [CI], 1.6 to 4.3) decrease in eGFR levels from baseline in SGLT2i users while a 1.7% (95% CI, 0.5 to 3.8) increase in eGFR levels was seen in nonusers. In an unadjusted linear mixed-effects model, patients with SGLT2is had a slightly higher mean total and chronic eGFR slope by 0.44 (95% CI, −0.44 to 1.31) and 1.46 (95% CI, 0.71 to 2.21) ml/min per 1.73 m^2^ per year, respectively, during the 2-year follow-up, compared with non-SGLT2i users (*P* = 0.07 and *P* < 0.001, respectively). An adjusted linear mixed-effect model has shown that total slope for non-SGLT2i users was −0.16 (95% CI, −3.31 to 2.99) ml/min per 1.73 m^2^ per year, compared with +0.039 (95% CI, −3.78 to 3.86) ml/min per 1.73 m^2^ per year, with a difference of 0.20 (95% CI, −0.46 to 0.86) ml/min per 1.73 m^2^ per year (*P* = 0.55). The postinitial dip slope for non-SGLT2i users was −0.66 (95% CI, −4.35 to 3.04) ml/min per 1.73 m^2^ per year, compared with 0.57 (95% CI, −3.91 to 5.06) ml/min per 1.73 m^2^ per year, with a significant difference of 1.23 (95% CI, 0.43 to 2.02) ml/min per 1.73 m^2^ per year, indicating a significantly slower eGFR decline in SGLT2i users than that in nonusers (*P* = 0.002). Among patients with available eGFR values at 1 month after SGLT2i initiation, the difference in total slope between SGLT2i users and nonusers was 0.30 ml/min per 1.73 m^2^ per year (*P* = 0.54), whereas that in slope from 1 month to 24 months after SGLT2i initiation was 1.4 ml/min per 1.73 m^2^ per year (*P* = 0.045; Supplemental Figure 1).

We matched 110 nonusers and 109 users of SGLT2i on the basis of their PSs. After matching, mean levels of initial eGFR dip in SGLT2i users were 2.5% (95% CI, 0.8 to 4.2); it was significantly decreased compared with nonusers (*P* = 0.001; Figure 2B). There were no statistical difference between users and nonusers of SGLT2i in total slope, with a difference of 0.52 (95% CI, 0.17 to 1.22) ml/min per 1.73 m^2^ per year (*P* = 0.14). SGLT2i users have a significantly lower postinitial dip slope than nonusers, 0.14 (95% CI, −4.48 to 4.75) among users and 1.63 (95% CI, −3.85 to 7.11) ml/min per 1.73 m^2^ per year among nonusers: The difference denoted a statistical significance of 1.50 (95% CI, 0.63 to 2.36) ml/min per 1.73 m^2^ per year (*P* < 0.001).

In all patients, comparison between SGLT2i users and nonusers revealed that SGLT2i was significantly associated with a longitudinal increase in hemoglobin concentration (*P* = 0.028) and BUN (*P* = 0.003) and a decrease in serum uric acid levels (*P* < 0.001). By contrast, no significant differences were observed between the groups with respect to electrolyte levels or urinary protein excretion (*P* = 0.84; Supplemental Figure 2).

### Subgroup Analyses

**Figure 3:**
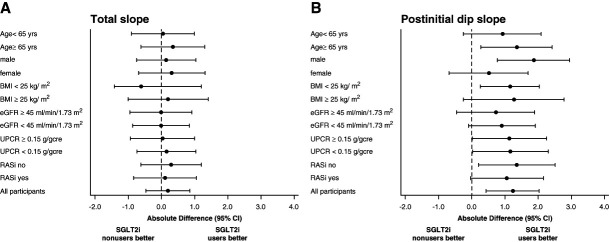
**Mean annual rate of eGFR changes in total slope and postinitial dip slope in subgroups.** (A) Total slope (B) postinitial dip slope. BMI, body mass index; CI, confidence interval; RASi, renin-angiotensin system inhibitor; UPCR, urinary protein–creatinine ratio.

Subgroup analyses of total and postinitial dip slopes were evaluated as shown in Figure 3, A and B, respectively. In total slope, the rates of eGFR decline in SGLT2i users and nonusers were likely to be almost similar. On the other hand, patients with SGLT2is significantly ameliorated their eGFR declines compared with those without SGLT2is in postinitial dip slope. The eGFR slope significantly improved, particularly in elderly individuals, men, those with a lean body type, and those not taking RASis. Furthermore, the effect of SGLT2is was observed regardless of whether proteinuria was below or above 0.15 g/gcr.

## Discussion

We highlight that the use of SGLT2is was associated with a significant improvement in eGFR decline in non-diabetic CKD patients with UPCR <0.5 g/gcre. There was no difference in the total slope between SGLT2i users and nonusers over the entire study period, but the annual change rate of eGFR significantly slowed down in postinitial dip slope after the initial dip of SGLT2is. After PS matching, this trend did not remain attenuated. Furthermore, subgroup analyses revealed SGLT2i use significantly slowed eGFR decline in patients both with UPCR <0.15 and 0.15–0.5 g/gcre. SGLT2is had beneficial effects on kidney protection in nonproteinuric or less proteinuric patients with CKD, demonstrating the robustness of our results.

Evidence of kidney-protective effects on SGLT2is has been progressively developed in diabetic patients,^7,9,10^ patients with CKD,^11,16^ and patients with heart failure (HF).^17–20^ The subanalysis in EMPA-REG OUTCOME showed that SGLT2is can slow down the decline of kidney function in diabetic patients without proteinuria and those with proteinuria.^13^ Moreover, patients with HF, which typically does not present proteinuria, experienced not only better cardiovascular outcomes, but also improved kidney outcomes with the addition of SGLT2is to renin-angiotensin system blockers.^21,22^ These beneficial effects have been equally provided in both HF with preserved ejection fraction and HF with reduced ejection fraction, suggesting its potential as a cardiorenal therapy.

On the other hand, in the CKD field, favorable effects on kidney deterioration have been clearly demonstrated for patients with proteinuria,^11,16^ but it is still unclear among patients without proteinuria. In patients with diabetes, both large-scale observational studies and small randomized controlled trials have reported that SGLT2is can decelerate the progression of kidney function decline, even in those with minimal proteinuria.^23,24^ However, there have been some subanalyses that implicated beneficial effects in nondiabetic patients without proteinuria. In DAPA-CKD, 25% risk reduction in the primary end point was achieved in the dapagliflozin group with hypertensive and ischemic nephropathy, without significant interactions observed with GN or diabetes.^12^ EMPA-KIDNEY revealed that, although empagliflozin versus placebo in nonproteinuric patients with CKD did not reach significance for kidney events and cardiovascular death, empagliflozin slowed both rates of total and postinitial dip eGFR slopes; the differences (95% CI) were 0.75 (0.54 to 0.96) and 1.37 (1.16 to 1.59) ml/min per 1.73 m^2^, respectively.^14^ Real-world data from OPTIMISE also showed a significant slope improvement of −1.28 (95% CI, 1.56 to 4.12) ml/min per 1.73 m^2^ in patients with UACR <200 mg/gcre without diabetes.^25^ We found similar effects of SGLT2is were observed even in patients with UPCR <0.15 g/gcre. Taken together with these results, our findings support a potential benefit of SGLT2is in nonproteinuric patients with CKD.

Mechanisms underlying kidney-protective effects of SGLT2is remained fully unknown in nonproteinuric CKD. SGLT2is modulate high glomerular pressure by afferent arteriole vasoconstriction, resulting in the reduction of proteinuria, but it is unclear to what extent this action affects conditions that are not primary glomerular diseases.^26^ SGLT2is also attenuate tubular injury by regulation of autophagy and endoplasmic reticulum stress and alteration of fuel source for increase in ketone bodies.^27–29^ Clinically, SGLT2is promote natriuresis and glucosuria, leading to appropriate control of night BP, body weight, and uric acid.^30–32^ SGLT2is prevent the occurrence of AKI and hyperkalemia, possibly contributing to facilitate persistent use of RASis.^33–35^

Several limitations were noted in this study. First, this investigation is a single-center observational study with a small sample size. Second, some variables associated with the use of SGLT2is may not be evaluated. Third, the number and frequency of eGFR measurements during the study period was limited. Fourth, chronic GN patients in this study predominantly consists of individuals with chronic lesions after treatment with steroids and immunosuppressive agents. Consequently, proteinuria is less pronounced compared with what has been reported in previous studies. Fifth, modifications in the use of renoprotective agents, including RASis and mineral corticoid receptor antagonists, over the course of the study were not incorporated into this analysis. The strength of our study lies in the robustness of our findings, derived after adjusting patient backgrounds using PS matching to minimize biases as much as possible, and in the novel observation that these results were achieved even among patients who had undergone comprehensive multidisciplinary CKD education.

In conclusion, beneficial effects of SGLT2is on eGFR decline in nondiabetic CKD with less proteinuria can be confirmed, as well as with proteinuria. Our results suggest the SGLT2i indication as kidney-protective agents might be expanded to include early-stage CKD without proteinuria.

## Supplementary Material

## Data Availability

Partial restrictions to the data and/or materials apply. Deidentified data is available upon reasonable request to the corresponding author.

## Supplemental Material

Supplemental Table 1. Other laboratory findings in SGLT2i users and nonusers.

Supplemental Figure 1. eGFR slope in patients with available eGFR values 1 month after SGLT2i initiation.

Supplemental Figure 2. Changes in clinical laboratory parameters before and after SGLT2i administration.
