# Supplementary material for: Effects of Sodium-Glucose Cotransporter 2 Inhibitors on Lower eGFR Decline in Nondiabetic CKD Patients without Proteinuria
Source: Kidney360. 2025 Jun 19;6(11):1899–905. doi: 10.34067/KID.0000000886 (PMC12626659; doi:10.34067/KID.0000000886)
Supplement: Supplementary file 1 [file kidney360-6-1899-s001.pdf]

ASN American Society of Nephrology
JASN Journal of the American Society of Nephrology
KIDNEY360 Accessing Our World From Every Angle
CJASN Clinical Journal of the American Society of Nephrology

ASN Journal Disclosure Form

As per ASN journal policy, I have disclosed any financial relationships or commitments I have held in the past 36 months as included below. I have listed my Current Employer below to indicate there is a relationship requiring disclosure. If no relationship exists, my Current Employer is not listed.

M. Eriguchi reports the following:
Employer: Nara medical University

I understand that the information above will be published within the journal article, if accepted, and that failure to comply and/or to accurately and completely report the potential financial conflicts of interest could lead to the following: 1) Prior to publication, article rejection, or 2) Post-publication, sanctions ranging from, but not limited to, issuing a correction, reporting the inaccurate information to the authors' institution, banning authors from submitting work to ASN journals for varying lengths of time, and/or retraction of the published work.

Name: Masahiro Eriguchi
Manuscript ID: K360-2025-000171R1
Manuscript Title: Effects of SGLT2 inhibitors on lower eGFR decline in non-diabetic CKD patients without proteinuria
Date of Completion: May 19, 2025
Disclosure Updated Date: May 19, 2025

ASN Journal Disclosure Form

As per ASN journal policy, I have disclosed any financial relationships or commitments I have held in the past 36 months as included below. I have listed my Current Employer below to indicate there is a relationship requiring disclosure. If no relationship exists, my Current Employer is not listed.

S. Kitamura reports the following:
Employer: Department of Nephrology, Nara Medical University

I understand that the information above will be published within the journal article, if accepted, and that failure to comply and/or to accurately and completely report the potential financial conflicts of interest could lead to the following: 1) Prior to publication, article rejection, or 2) Post-publication, sanctions ranging from, but not limited to, issuing a correction, reporting the inaccurate information to the authors' institution, banning authors from submitting work to ASN journals for varying lengths of time, and/or retraction of the published work.

Name: Shunsuke Kitamura
Manuscript ID: K360-2025-000171R1
Manuscript Title: Effects of SGLT2 inhibitors on lower eGFR decline in non-diabetic CKD patients without proteinuria
Date of Completion: May 19, 2025
Disclosure Updated Date: May 10, 2025

ASN American Society of Nephrology
JASN Journal of the American Society of Nephrology
KIDNEY360 Accessing Our World From Every Angle
CJASN Clinical Journal of the American Society of Nephrology

ASN Journal Disclosure Form

As per ASN journal policy, I have disclosed any financial relationships or commitments I have held in the past 36 months as included below. I have listed my Current Employer below to indicate there is a relationship requiring disclosure. If no relationship exists, my Current Employer is not listed.

T. Kosugi reports the following:
Honoraria: Kowa, Torii, Sanwa Kagaku Kenkyusho, and AstraZeneca

I understand that the information above will be published within the journal article, if accepted, and that failure to comply and/or to accurately and completely report the potential financial conflicts of interest could lead to the following: 1) Prior to publication, article rejection, or 2) Post-publication, sanctions ranging from, but not limited to, issuing a correction, reporting the inaccurate information to the authors' institution, banning authors from submitting work to ASN journals for varying lengths of time, and/or retraction of the published work.

Name: Takaaki Kosugi
Manuscript ID: K360-2025-000171R1
Manuscript Title: Effects of SGLT2 inhibitors on lower eGFR decline in non-diabetic CKD patients without proteinuria
Date of Completion: May 19, 2025
Disclosure Updated Date: May 19, 2025

ASN American Society of Nephrology
JASN Journal of the American Society of Nephrology
KIDNEY360 Accessing Our World From Every Angle
CJASN Clinical Journal of the American Society of Nephrology

ASN Journal Disclosure Form

As per ASN journal policy, I have disclosed any financial relationships or commitments I have held in the past 36 months as included below. I have listed my Current Employer below to indicate there is a relationship requiring disclosure. If no relationship exists, my Current Employer is not listed.

M. Matsui reports the following:
Employer: Nara Medical Universuty

I understand that the information above will be published within the journal article, if accepted, and that failure to comply and/or to accurately and completely report the potential financial conflicts of interest could lead to the following: 1) Prior to publication, article rejection, or 2) Post-publication, sanctions ranging from, but not limited to, issuing a correction, reporting the inaccurate information to the authors' institution, banning authors from submitting work to ASN journals for varying lengths of time, and/or retraction of the published work.

Name: Masaru Matsui
Manuscript ID: K360-2025-000171R1
Manuscript Title: Effects of SGLT2 inhibitors on lower eGFR decline in non-diabetic CKD patients without proteinuria
Date of Completion: May 19, 2025
Disclosure Updated Date: May 19, 2025

ASN American Society of Nephrology
JASN Journal of the American Society of Nephrology
KIDNEY360 Accessing Our World From Every Angle
CJASN Clinical Journal of the American Society of Nephrology

ASN Journal Disclosure Form

As per ASN journal policy, I have disclosed any financial relationships or commitments I have held in the past 36 months as included below. I have listed my Current Employer below to indicate there is a relationship requiring disclosure. If no relationship exists, my Current Employer is not listed.

M. Nishimoto reports the following:
Employer: Nara Medical University

I understand that the information above will be published within the journal article, if accepted, and that failure to comply and/or to accurately and completely report the potential financial conflicts of interest could lead to the following: 1) Prior to publication, article rejection, or 2) Post-publication, sanctions ranging from, but not limited to, issuing a correction, reporting the inaccurate information to the authors' institution, banning authors from submitting work to ASN journals for varying lengths of time, and/or retraction of the published work.

Name: Masatoshi Nishimoto
Manuscript ID: K360-2025-000171R1
Manuscript Title: Effects of SGLT2 inhibitors on lower eGFR decline in non-diabetic CKD patients without proteinuria
Date of Completion: May 19, 2025
Disclosure Updated Date: May 19, 2025

ASN American Society of Nephrology | JASN Journal of the American Society of Nephrology | KIDNEY360 Accessing Our World From Every Angle | CJASN Clinical Journal of the American Society of Nephrology

ASN Journal Disclosure Form

As per ASN journal policy, I have disclosed any financial relationships or commitments I have held in the past 36 months as included below. I have listed my Current Employer below to indicate there is a relationship requiring disclosure. If no relationship exists, my Current Employer is not listed.

K. Okamoto reports the following:
Employer: Nara Medical University

I understand that the information above will be published within the journal article, if accepted, and that failure to comply and/or to accurately and completely report the potential financial conflicts of interest could lead to the following: 1) Prior to publication, article rejection, or 2) Post-publication, sanctions ranging from, but not limited to, issuing a correction, reporting the inaccurate information to the authors' institution, banning authors from submitting work to ASN journals for varying lengths of time, and/or retraction of the published work.

Name: Keisuke Okamoto
Manuscript ID: K360-2025-000171R1
Manuscript Title: Effects of SGLT2 inhibitors on lower eGFR decline in non-diabetic CKD patients without proteinuria
Date of Completion: May 19, 2025
Disclosure Updated Date: May 19, 2025

ASN American Society of Nephrology
JASN Journal of the American Society of Nephrology
KIDNEY360 Accessing Our World From Every Angle
CJASN Clinical Journal of the American Society of Nephrology

ASN Journal Disclosure Form

As per ASN journal policy, I have disclosed any financial relationships or commitments I have held in the past 36 months as included below. I have listed my Current Employer below to indicate there is a relationship requiring disclosure. If no relationship exists, my Current Employer is not listed.

K. Samejima reports the following:
Employer: Department of Nephrology, Nara Medical University

I understand that the information above will be published within the journal article, if accepted, and that failure to comply and/or to accurately and completely report the potential financial conflicts of interest could lead to the following: 1) Prior to publication, article rejection, or 2) Post-publication, sanctions ranging from, but not limited to, issuing a correction, reporting the inaccurate information to the authors' institution, banning authors from submitting work to ASN journals for varying lengths of time, and/or retraction of the published work.

Name: Ken-ichi Samejima
Manuscript ID: K360-2025-000171R1
Manuscript Title: Effects of SGLT2 inhibitors on lower eGFR decline in non-diabetic CKD patients without proteinuria
Date of Completion: May 21, 2025
Disclosure Updated Date: May 21, 2025

ASN American Society of Nephrology | JASN Journal of the American Society of Nephrology | KIDNEY360 Accessing Our World From Every Angle | CJASN Clinical Journal of the American Society of Nephrology

ASN Journal Disclosure Form

As per ASN journal policy, I have disclosed any financial relationships or commitments I have held in the past 36 months as included below. I have listed my Current Employer below to indicate there is a relationship requiring disclosure. If no relationship exists, my Current Employer is not listed.

K. Tansho reports the following:
Employer: Nara prefecture General Medical Center

I understand that the information above will be published within the journal article, if accepted, and that failure to comply and/or to accurately and completely report the potential financial conflicts of interest could lead to the following: 1) Prior to publication, article rejection, or 2) Post-publication, sanctions ranging from, but not limited to, issuing a correction, reporting the inaccurate information to the authors' institution, banning authors from submitting work to ASN journals for varying lengths of time, and/or retraction of the published work.

Name: Kosuke Tansho
Manuscript ID: K360-2025-000171R1
Manuscript Title: Effects of SGLT2 inhibitors on lower eGFR decline in non-diabetic CKD patients without proteinuria
Date of Completion: May 19, 2025
Disclosure Updated Date: May 19, 2025

ASN American Society of Nephrology · JASN Journal of the American Society of Nephrology · KIDNEY360 Accessing Our World From Every Angle · CJASN Clinical Journal of the American Society of Nephrology

ASN Journal Disclosure Form

As per ASN journal policy, I have disclosed any financial relationships or commitments I have held in the past 36 months as included below. I have listed my Current Employer below to indicate there is a relationship requiring disclosure. If no relationship exists, my Current Employer is not listed.

K. Tsuruya reports the following:
Employer: Nara Medical University; Research Funding: Baxter; Bayer; Boehringer Ingelheim; Daiichi-Sankyo; Kissei; Kyowa Kirin; Mitsubishi Tanabe; Otsuka; Teijin; Terumo; Torii; Honoraria: Astellas; AstraZeneca; Baxter; Bayer; Boehringer Ingelheim; Bristol Myers Squibb; Chugai; Daiichi-Sankyo; Fuso; GSK; Kaneka; Kissei; Kowa; Kyowa Kirin; Mitsubishi Tanabe; Mochida; MSD; Novartis; Mylan EPD; Ono; Otsuka; Sanofi; Sanwa Chemistry; Sumitomo Dainippon; Teijin; Terumo; Torii; Advisory or Leadership Role: Astellas; AstraZeneca; Mitsubishi Tanabe; Kyowa Kirin; and Speakers Bureau: Astellas; AstraZeneca; Baxter; Bayer; Boehringer Ingelheim; Fuso; GSK; Kaneka; Kissei; Kowa; Kyowa Kirin; Mitsubishi Tanabe; Mochida; MSD; Novartis; Ono; Otsuka; Sanofi; Sanwa Chemistry; Sumitomo Pharma; Teijin; Terumo; Torii.

I understand that the information above will be published within the journal article, if accepted, and that failure to comply and/or to accurately and completely report the potential financial conflicts of interest could lead to the following: 1) Prior to publication, article rejection, or 2) Post-publication, sanctions ranging from, but not limited to, issuing a correction, reporting the inaccurate information to the authors' institution, banning authors from submitting work to ASN journals for varying lengths of time, and/or retraction of the published work.

Name: Kazuhiko Tsuruya
Manuscript ID: K360-2025-000171R1
Manuscript Title: Effects of SGLT2 inhibitors on lower eGFR decline in non-diabetic CKD patients without proteinuria
Date of Completion: May 19, 2025
Disclosure Updated Date: May 11, 2025
