# Supplementary material for: Effects of Sodium-Glucose Cotransporter 2 Inhibitors on Lower eGFR Decline in Nondiabetic CKD Patients without Proteinuria
Source: Kidney360. 2025 Jun 19;6(11):1899–905. doi: 10.34067/KID.0000000886 (PMC12626659; doi:10.34067/KID.0000000886)

**Supplemental Table 1. Other Laboratory Findings in SGLT2i users and non-users**

| | SGLT2i users | non SGLT2i users | P value |
|---|---|---|---|
| No. of participants | 211 | 151 | |
| Uric acid, mg/dL | 6.16 (5.05–7.12) | 5.96 (5.13–6.81) | 0.46 |
| Urea, mg/dL | 19.3 (15.8-23.4) | 19.5 (14.4-24.5) | 0.37 |
| Creatinine, mg/dL | 1.18 (1.01-1.44) | 1.22 (0.97-1.49) | 0.54 |
| Sodium, mEq/L | 140 (139-142) | 140 (139-142) | 0.41 |
| Potassium, mEq/L | 4.2 (4.0-4.5) | 4.3 (4.0-4.5) | 0.09 |
| Chloride, mEq/L | 103 (102-105) | 105 (104-107) | <0.001 |

Abbreviations: SGLT2i, sodium-glucose cotransporter 2 inhibitors

**Supplemental Figure 1: eGFR slope in patients with available eGFR values one month after SGLT2i initiation**

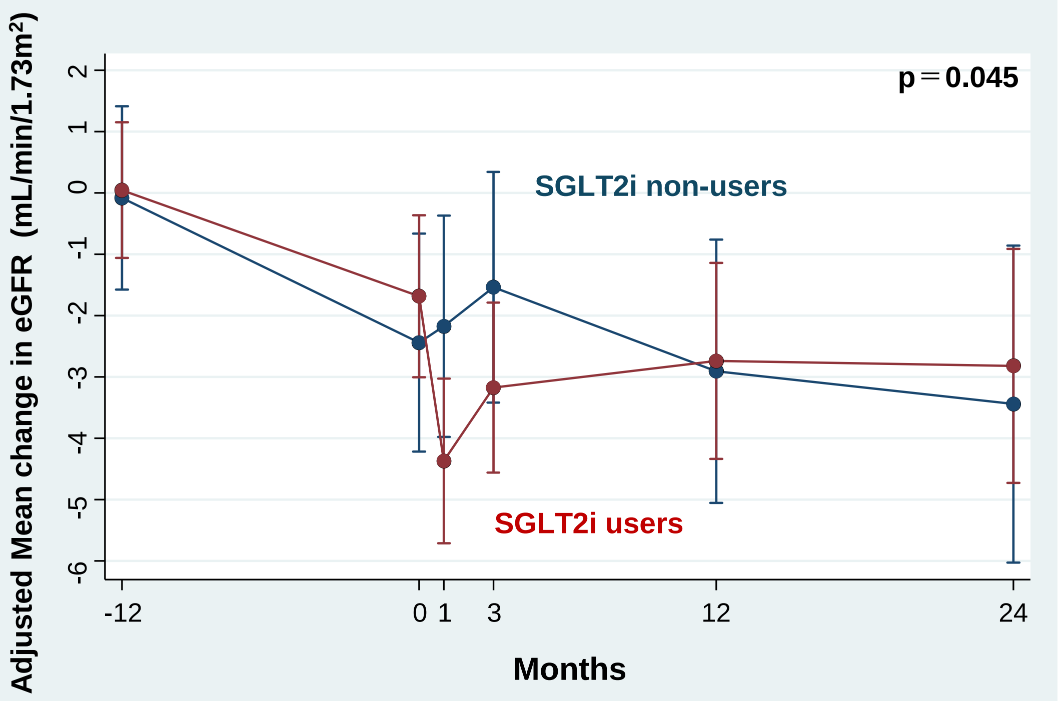


| | | | | | | |
|---|---|---|---|---|---|---|
| SGLT2i users | 98 | 98 | 98 | 98 | 97 | 96 |
| non-users | 54 | 54 | 54 | 52 | 53 | 50 |

**Supplemental Figure 2: Changes in Clinical Laboratory Parameters Before and After SGLT2 Inhibitor Administration**

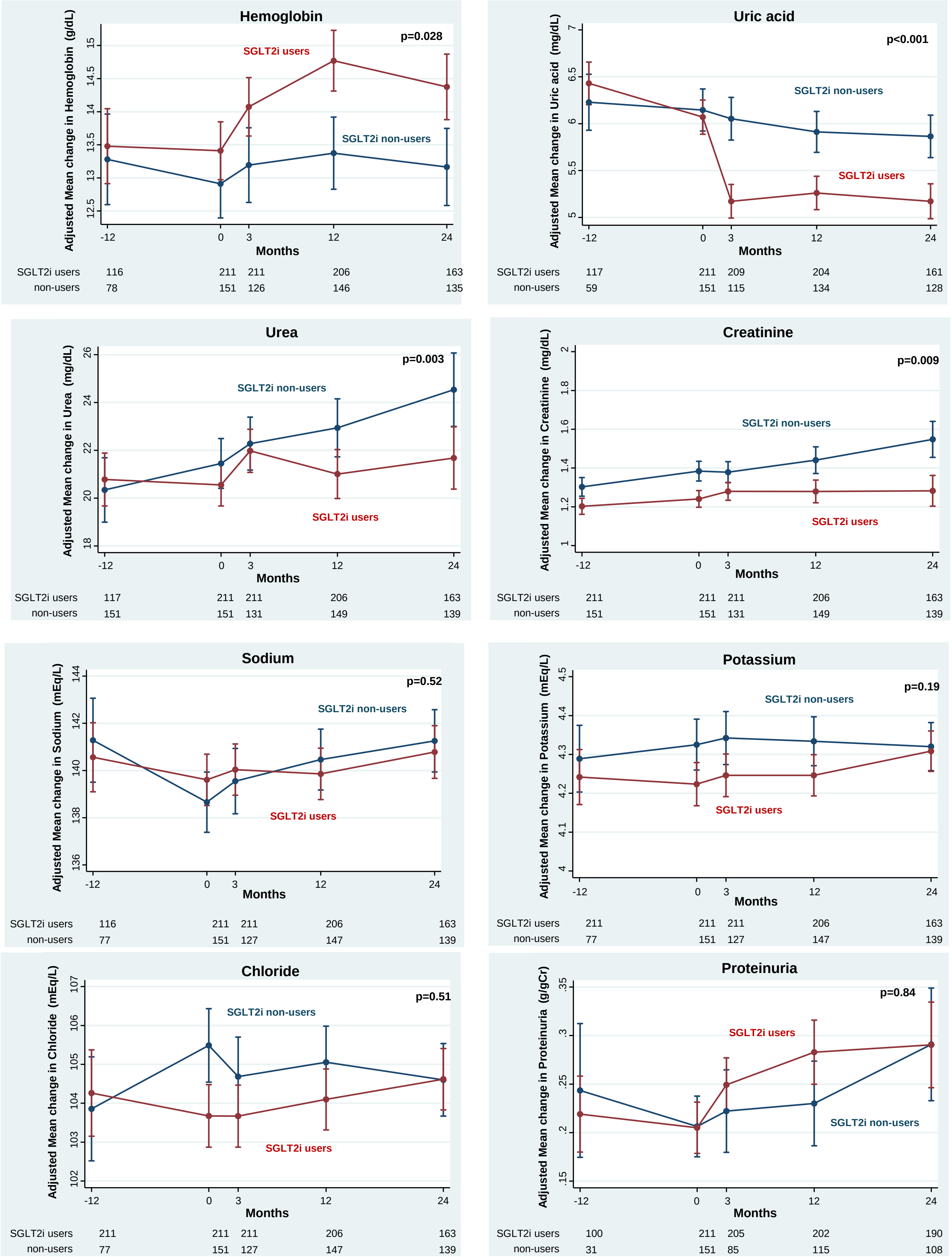

Supplement: Supplementary file 2 [file kidney360-6-1899-s002.pdf]
